# Risk factors, physical activity behavior, and hand grip strength of adults with atherosclerotic cardiovascular disease in Arar city, Saudi Arabia: A cross-sectional study

**DOI:** 10.1097/MD.0000000000045977

**Published:** 2025-11-28

**Authors:** Mansour M. Alotaibi, Anwar B. Almutairi, Manal A. Alhowaish, Mohammed M. Alqahtani, Marzouq K. Almutairi, Abubakr S. Siddig, Wajdan O. Alenazi, Amina A. Alenazi, Ansar S. Albanagi, Aisha S. Alenazi, Naif Z. Alrashdi

**Affiliations:** a Department of Rehabilitation, College of Applied Medical Sciences. Northern Border University, Arar, Northern Border Province, Saudi Arabia; b Center for Health Research, Northern Border University, Arar, Saudi Arabia; c Department of Physical Therapy, Faculty of Allied Health, Kuwait University, Jabriya, Kuwait; d Department of Critical Care Unit, Prince Abdullah Bin Abdulaziz Bin Musaed of Cardiac Center, Arar, Saudi Arabia; e Department of Respiratory Therapy, College of Applied Medical Sciences, King Saud bin Abdulaziz University for Health Sciences, Riyadh, Saudi Arabia; f King Abdullah International Medical Research Center, Riyadh, Saudi Arabia; g Department of Respiratory Services, King Abdulaziz Medical City, Ministry of National Guard Health Affairs, Riyadh, Saudi Arabia; h Department of Physical Therapy, College of Applied Medical Sciences, Qassim University, Buraidah, Qassim Province, Saudi Arabia; i Department of Cardiology, Dr Sulaiman Al Habib Medical Group, Al Muhmmadiyah Hospital, Jeddah, Saudi Arabia; j Department of Cardiovascular Intensive Care Unit, Prince Abdullah Bin Abdulaziz Bin Musaed of Cardiac Center, Arar, Saudi Arabia; k Department of Emergency, North Medical Tower, Arar, Saudi Arabia; l Department of Cardiac Catheterization, Prince Abdullah Bin Abdulaziz Bin Musaed of Cardiac Center, Arar, Saudi Arabia; m Department of Physical Therapy and Health Rehabilitation, College of Applied Medical Sciences, Majmaah University, Al-Majmaah, Saudi Arabia; n Health and Basic Sciences Research Center, Majmaah University, Al-Majmaah, Saudi Arabia.

**Keywords:** cardiac, Godin, left ventricle ejection fraction, muscle strength, physical activity

## Abstract

Cardiovascular diseases (CVD) remain the leading cause of global mortality. Risk factors such as diabetes mellitus, hypertension, dyslipidemia, smoking, low physical activity, weak handgrip strength, and high body mass index are commonly associated with CVD. This study explores risk factors, physical activity behaviors, and handgrip strength in adults diagnosed with atherosclerotic CVD in Arar city, Saudi Arabia. A cross-sectional analysis was conducted with 100 patients with CVD admitted to a local cardiac center between August 2023 and April 2024. Participants completed the Godin Leisure-Time Exercise Questionnaire to assess physical activity. Handgrip strength was measured using a handgrip strength dynamometer. Additional clinical, demographic, and anthropometric data, including left ventricle ejection fraction (LVEF) and hemoglobin A1c levels, were retrieved from the center’s records. Differences in physical activity and handgrip strength between diabetes groups (normal, prediabetic, and diabetic) and LVEF groups (normal LVEF, mildly reduced LVEF, and reduced LVEF) were analyzed using analysis of variance, while setting *P* < .05 as the criterion for significant difference. Of the participants (88% men, mean age 55.6 ± 11.9 years), 73% were insufficiently active, as per the Godin Leisure-Time Exercise Questionnaire score. Handgrip strength and physical activity scores were higher among non-diabetic and normal LVEF groups compared to the other groups. Nonetheless, differences between groups were not statistically significant (*P* > .05). Most patients exhibited common CVD risk factors, such as increased body mass index, dyslipidemia, and smoking history, with 36% having single vessel disease and 38% reduced LVEF. This study highlights low physical activity levels and weak handgrip strength in CVD patients in Arar city, highlighting a critical need for tailored public health initiatives. Programs promoting physical activity and resistance training may enhance cardiac health and mitigate risk. Region-specific insights inform national strategies to address reducing the risk of CVD in Saudi Arabia.

## 1. Introduction

Cardiovascular disease (CVD) is a leading cause of mortality with an estimate of 17.9 million deaths from CVD in 2019, constituting approximately one-third of all global deaths.^[1]^ More than 30% of adults aged 18 years or older are at high risk of developing CVD events.^[2,3]^ Risk factors that are known to associate with CVD include diabetes mellitus (DM), hypertension, dyslipidemia, smoking, and high body mass index (BMI),^[4,5]^ all of which contribute substantially to cardiovascular attributable mortality.^[4]^ Current evidence shows that high-income countries have higher rates of CVD diseases compared to low-income countries.^[4]^ In Saudi Arabia, CVD is identified as a national health priority that is aligned with the Saudi Vision 2030 strategic goal of increasing life expectancy from 75 to 80 years.^[5]^ Despite national initiatives,^[5]^ there remains a need for region-specific data to guide prevention and treatment strategies. Previous research in Arar city highlighted the prevalence of obesity and smoking as potential contributors to CVD,^[6,7]^ yet little is known about how physical activity levels and muscle strength are related to cardiac function in this population.

Monitoring demographic, anthropometric, behavioral, and clinical factors such as age, smoking status, and cardiac blood markers, such as troponin I enzyme and creatine kinase (CK), are important factors for development of region-specific and national CVD prevention strategies. These factors are often used to identify those at risk of CVD through scoring mechanisms, such as the Framingham Risk Score.^[8]^ Therefore, determining regional-specific risk factors is important for developing new prevention and treatment strategies and may help current and future national programs in mitigating the current increased risk of CVD.^[2,3]^

Therefore, this study aimed to describe CVD risk factors, physical activity behaviors, and handgrip strength among patients admitted to Prince Abdullah Bin Musaed Cardiac Centre (PAMCC) in Arar city. A secondary aim was to examine differences in physical activity and grip strength by diabetes status and left ventricular ejection fraction (LVEF). We hypothesized that patients with CVD would exhibit elevated risk factors (e.g., high BMI, dyslipidemia, hypertension, and low physical activity), and that physical activity and grip strength would be higher in non-diabetic patients with normal LVEF compared to other subgroups.

## 2. Methods

### 2.1. Study design and participants

This study used a cross-sectional study design to characterize patients with CVD that were admitted to PAMCC in Arar city, Saudi Arabia. CVD is defined as a set of disorders of the heart and blood vessels, including coronary heart disease, cerebrovascular disease, peripheral arterial disease, rheumatic heart disease, congenital heart disease, and deep vein thrombosis and pulmonary embolism.^[9]^ We enrolled a sample consisting of 100 patients with CVD and data collection started in August 2023 and continued until April 2024. To be included, participants had to be diagnosed with CVD by a cardiologist confirmed by coronary angiogram (CAG) report, admitted to PAMCC for CVD-related emergency events or symptoms (e.g., severe chest pain, cardiac arrhythmia, and shoulder pain of the left side), or referred from other healthcare facilities in the region or presentation as urgent cases at PAMCC. The exclusion criteria were: participants with cognitive dysfunction impairing their ability to follow instructions and participants that had severe orthopedic injury that affected their ability to engage in physical activity. All participants provided informed written consent and our study protocol was approved by the bioethical committee at Northern Border University (protocol number: HAP-09-A-043).

### 2.2. Procedures

Upon admission, participants were transferred directly to the cardiac catheterization room at PAMCC for immediate treatment (standard care provided at PAMCC). After the patient status stabilizes (at least 72 hours post the last CAG procedure), participants provided information regarding their physical activity level by answering questions of the Goden Leisure-Time Exercise Questionnaire (GLTEQ).^[10]^ Finally, participants completed 3 trials of hand grip strength using the Takei T.K.K 5401 digital dynamometer (Takei Scientific Instruments Co. Ltd., Tokyo, Japan) for the hand that was free from the CAG procedure with a 60-second rest time provided between the trials. Additional variables were retrieved from the medical system, including main diagnosis, demographic variables (age, sex, and nationality), anthropometric variables, CAG classification (plaque of single or multiple vessels), CAG intervention (e.g., stent), major risk factors (hyperlipidemia, diabetes, and hypertension), cardiac and other blood biomarkers (e.g., troponin I), LVEF, and medication information. After the patient status stabilizes (at least 72 hours post the last CAG procedure). In addition, participants provided information regarding their physical activity level by answering questions of the GLTEQ. Finally, participants completed 3 trials of hand grip strength using the Takei T.K.K 5401 digital dynamometer (Takei Scientific Instruments Co. Ltd., Tokyo, Japan) for the hand that was free from the CAG procedure with a 60-second rest period provided between the trials.

### 2.3. Outcome measures

#### 2.3.1. Demographics, smoking, and anthropometric measurements

After consenting, participants completed several demographic-related questions including age, sex, nationality, educational level based on the highest degree obtained, residence (rural vs urban areas), and smoking behaviors (current smoker, previous smoker, or never smoked).^[11]^ Participants self-reported their body mass (kg) and body height (cm), and BMI was calculated as k/m^2^.

#### 2.3.2. Cardiac profile

The main diagnosis of patients was identified by a cardiologist and confirmed by the CAG report. Further information about atherosclerosis was also collected, including blockage type of the coronary artery (i.e., no block; single, double, or triple vessel disease). In addition, we recorded the type of intervention delivered to patients upon admission, including medication only, percutaneous coronary intervention, percutaneous transluminal coronary angioplasty, or coronary artery bypass grafting. We also documented the type of CAG intervention (e.g., ballon or stent), if applied. Furthermore, LVEF was measured by an experienced radiology technician using Philips CX-50 xMATRIX echocardiography device (Philips Healthcare, Andover). Patients were further classified to have normal LVEF (≥50%), mildly reduced LVEF (41–49%), or reduced LVEF (≤40%) as defined by the American Heart Association (AHA).^[12]^

#### 2.3.3. Blood markers

Upon admission, blood samples were obtained from the patients as part of their routine clinical care at PAMCC (5–10 mL). The blood work involved lipid profile (total cholesterol, high-density lipid protein, HDL; low-density protein [LDL]; triglycerides), as part of the cardiovascular disease screening and risk assessment.^[13]^ Further, glycated hemoglobin (hemoglobin A1c [HbA1c]) was also assessed as its value is independently associated with risk of coronary artery atherosclerosis.^[14]^ Finally, cardiac blood markers (troponin I enzyme test; CK serum; CK myocardial band serum) were retrieved from the patients’ records as they were part of assessment at PMACC.^[15]^ All of these tests were recommended by the AHA as a screening component for coronary artery disease risk.^[11]^

#### 2.3.4. Handgrip strength

The handgrip strength test assessed the isometric hand grip strength (kg) using the Takei Digital Grip Strength Dynamometer (Model T.K.K.5401; Takei Medical). The Takei Digital Grip Strength Dynamometer has been used in several national health surveys, such as the NHANES^[16]^ and the Korean National Health and Nutrition Examination Survey.^[17]^ To ensure consistency and reliability, all assessments were conducted by trained nurses at PAMCC who had undergone standardized training sessions in the use of the dynamometer. The examiner explained and demonstrated the test to participants. The examiner adjusted the grip size of the dynamometer to the hand size of each participant and then asked the participant to squeeze the dynamometer’s grip as much as they could (a practice trial). This practice trial was performed to ensure that the participant understood the testing procedure and the grip size was properly adjusted. Next, the examiner asked the participant to squeeze the dynamometer’s grip as hard as possible with 1 hand for about 3 to 5 seconds. Participants completed 3 trials with a 60-seconds rest that was allowed between trials. Participants alternated between hands to further increase rest time between trials. We selected the best grip strength performance for each hand (higher value) of the 3 trials and calculated the average grip strength score. Absolute and relative hand grip strength (maximum hand grip strength divided by BMI) were reported. Relative hand grip strength was found associated with cardiovascular profile in adults.^[18]^

#### 2.3.5. Physical activity – GLTEQ

The GLTEQ assesses the frequency of physical activity bouts of ≥ 15 minutes when performing mild, moderate, and strenuous intensity of physical activity.^[10]^ The GLTEQ score of moderate and strenuous physical activity provided an overall Health Contribution Score (HCS) that has been previously found to be reliable and aligns with the Physical Activity Guidelines for Americans (PAGA) in adults.,^[10]^ and has shown construct validity when used with healthy adults.^[10]^ The GLTEQ score is based on the frequency of self-reported light, moderate, and strenuous intensity exercise bouts only and multiplying these frequencies by weights of 3, 5, and 9 metabolic equivalents of task, respectively. The HCS excludes weights of light exercise bouts, yielding only scores of moderate and strenuous multiples that are summed and range between 0 and 98, and reflect 3 categories: insufficiently active (<14 units), moderately active (14–23 units), and active (≥ 24 units).^[10]^

### 2.4. Data processing and statistical analyses

The Statistical Package for Social Sciences software (SPSS; IBM Corp., Armonk) v29.0 guided the statistical analyses. Means and standard deviations were used to characterize all the continuous variables, whereas frequencies and relative frequencies described the categorical variables. Continuous variables include age (years), body mass (kg), body height (cm), BMI (kg/m^2^), total cholesterol (mmol/L), high-density lipid protein (mmol/L), LDL, (mmol/L), triglycerides (mmol/L), troponin I (ng/mL), CK (IU/L), CK-myocardial band (IU/L), HbA1c (%), LVEF (%), GLTEQ score (units), GLTEQ-HCS score (units), absolute grip strength (kg), and relative grip strength (kg/BMI). Categorical variables include sex, nationality, educational level, living in rural vs urban area, smoking status, income, main diagnosis, CAG intervention type, physical activity category by GLTEQ, having diabetes, and having reduced LVEF. Two 1-way analysis of variance models determined the differences in GLTEQ physical activity score (unit), absolute grip strength (kg), and relative grip strength (kg/BMI) between diabetes groups (normal, prediabetic, and diabetic), and between cardiac performance groups based on LVEF (i.e., normal LVEF, mildly reduced LVEF, and reduced LVEF). Diabetic group assignment was based on HbA1c levels (normal: <5.7%, prediabetic: between 5.7 and below 6.5%, and diabetic: ≥ 6.5%). Participants were further classified into normal LVEF (between 50–70%), mildly reduced LVEF (between 41% and 49%), or reduced LVEF (≤40%).^[12]^ post hoc Tukey tests were used to compare the differences among the previous groups if the analysis of variance models provided significant differences. A *P* value of .05 or less was considered significant. In addition, prior to considering multivariate modeling, we tested the assumptions of linearity and examined bivariate associations between physical activity (GLTEQ scores) and key predictors (age, sex, BMI, HbA1c, LVEF, smoking status, and grip strength). These tests revealed no significant relationships between the dependent variable and the predictors. Therefore, a multivariate model was not conducted, as it would not have added explanatory value.

## 3. Results

A total of 100 cardiac patients participated in our study with an average age of 55.6 ± 11.9 years. Approximately, 88% of were men, 61% were Saudis, and 71% had a history of smoking, including current and ex-smokers. Their average weight and height were 80.9 ± 17.9 (Kg) and 166.8 ± 7.2 (cm), respectively. Based on the coronary angiography, 35 patients had single vessel disease, 28 had double vessel disease, and 33 had triple vessel disease. The primary intervention provided was percutaneous coronary intervention in 77 patients. Out of 100 included participants, 73 were classified as insufficiently active based on GLTEQ scores. Demographic and clinical characteristics of the included sample are presented and summarized in Table 1.

**Table 1:** Participants characteristics by sex.

Variable	Male (n = 88)	Female (n = 12)	Overall (n = 100)
Age (yr)	54.8 (12.3)	60.9 (7.0)	55.6 (11.9)
Nationality n (%)			
Saudi	52 (59.1%)	9 (75.0%)	61 (61.0%)
West Asian	16 (18.2%)	2 (16.7%)	18 (18.0%)
North African	3 (3.4%)	0 (0.0%)	3 (3.0%)
Others	17 (19.3%)	1 (8.3%)	18 (18.0%)
Educational level n (%)			
High school	71 (80.7%)	1 (100.0%)	83 (83.0%)
Did some college/undergraduate	16 (18.2%)	0 (0.0%)	16 (16.0%)
Graduate level	1 (1.1%)	0 (0.0%)	1 (1.0%)
Place of living, n (%)			
City	79 (89.9%)	1 (100.0%)	91 (91.0%)
Governate or small town	9 (10.2%)	0 (0.0%)	9 (9.0%)
Smoking status n (%)			
Current smoker	17 (19.5%)	11 (91.7%)	50 (50.5%)
Ex-smoker	50 (57.5%)	0 (0.0%)	21 (21.2%)
Never smoked	20 (23.0%)	1 (8.3%)	28 (28.3%)
Body weight (kg)	80.8 (17.9)	82.0 (18.0)	80.9 (17.9)
Body height (cm)	167.5 (7.2)	161.2 (4.7)	166.8 (7.2)
Body mass index (kg/m^2^)	28.5 (5.5)	29.8 (7.0)	28.7 (5.7)
Income, n (%)			
>10,000 SAR/month	65 (74.7%)	12 (100.0%)	77 (77.8%)
<10,000 SAR/month	22 (25.3%)	0 (0.0%)	22 (22.2%)
CAG classification n (%)			
No blockage	1 (1.2%)	0 (0.0%)	1 (1.0%)
Single vessel disease	29 (34.1%)	6 (50.0%)	35 (36.1%)
Double vessel disease	24 (28.2%)	4 (33.3%)	28.0 (28.9)
Triple vessel disease	31 (36.5%)	2 (16.7%)	33 (34.0%)
CAG intervention type n (%)			
Medication only	63 (75.0%)	10 (83.3%)	12 (12.5%)
PCI/PCTA	14 (16.7%)	2 (16.7%)	77 (80.2%)
CABG	7 (8.3)	0 (0.0%)	7 (7.3%)
LVEF categories n (%)			
Normal	36 (41.4%)	8 (66.7%)	44 (44.4%)
Mildly reduced	16 (18.4%)	1 (8.3%)	17 (17.2%)
Reduced	35 (40.2%)	3 (25.0%)	38 (38.4%)
Total cholesterol (mmol/L)	7.6 (24.3)	4.7 (1.3)	7.3 (22.7)
High-density protein (mmol/L)	0.9 (0.2)	1.0 (0.2)	0.9 (0.2)
Low-density protein (mmol/L)	3.2 (1.9)	2.4 (1.7)	3.1 (1.9)
Triglycerides (mmol/L)	1.9 (1.2)	2.0 (1.1)	1.9 (1.2)
Troponin I (ng/L)	5.1 (10.4)	1.7 (2.5)	4.5 (9.7)
CK (U/L)	1104.8 (1159.4)	398.2 (564.6)	1012.6 (1123.7)
CK-MB (U/L)	234.7 (502.4)	68.8 (77.8)	214.0 (473.5)
HbA1c (%)	7.3 (2.0)	8.3 (2.3)	7.4 (2.1)
Diabetes status by HbA1c n (%)			
Normal	23 (28.0%)	2 (16.7%)	25 (26.6%)
Prediabetic	17 (20.7%)	1 (8.3%)	18 (19.1%)
Diabetic	42 (51.2%)	9 (75.0%)	51 (54.3%)
Physical activity			
GLTEQ (units)	20.5 (17.6)	15.4 (7.6)	19.8 (16.7)
GLTEQ – HCS (units)	9.8 (16.1)	4.1 (10.1)	9.2 (15.6)
GLTEQ categories n (%)			
Insufficiently active	62 (71.3%)	11 (91.7%)	73 (73.7%)
Moderately active	7 (8.0%)	0 (0.0%)	7 (7.1%)
Active	18 (20.7%)	1 (8.3%)	19 (19.2%)
Hand grip strength (kg)	32.8 (7.5)	18.5 (5.0)	31.0 (8.6)
Normalized hand grip strength (kg/BMI)	1.2 (0.3)	0.64 (0.2)	1.1 (0.3)

*Note*: Data are presented as means (and standard deviations) or numbers (and percentages).

CABG = coronary artery bypass grafting, CK = creatine kinase, CK-MB = creatine kinase myocardial band, GLTEQ = Godin Leisure-Time Exercise Questionnaire, HCS = Health Contribution Score, LPA = light physical activity, MVPA = moderate-to-vigorous physical activity, PCI = percutaneous coronary intervention.

Descriptive values (means) for the GLTEQ scores, GLTEQ-HSC, and grip strength by diabetes status are shown in Table 2. Our results showed that patients with normal HbA1c had higher GLTEQ and GLTEQ-HCS scores (22.6 ± 20.2 units, and 12.2 ± 18.2 units, respectively) compared to those who were prediabetic (20.2 ± 13.4 units, and 9.2 ± 13.8 units, respectively) or diabetic (19.1 ± 16.5 units and 8.3 ± 15.4 units). Additionally, patients with normal HbA1c exhibited higher relative grip strength (1.2 ± 0.4) than those with classified as prediabetes (1.1 ± 0.3 kg) or diabetes (1.0 ± 0.3 kg).

**Table 2:** Participant differences in physical activity score, absolute grip strength, and relative grip strength as classified by diabetes status.

Outcome measure	HbA1c diabetes status						*F* (2,88)	*η^2^*
	Normal		Prediabetic		Diabetic			
	M	SD	M	SD	M	SD		
Physical activity								
GLTEQ (units)	22.6	20.2	20.2	13.4	19.1	16.5	0.34	<0.01
GLTEQ-HCS (units)	12.2	18.2	9.2	13.8	8.3	15.4	0.51	0.01
Absolute hand grip strength (kg)	32.2	10.0	32.4	6.3	29.4	8.4	1.36	0.03
Relative grip strength (kg/BMI)	1.2	0.4	1.1	0.3	1.0	0.3	2.64	0.06

GLTEQ = Godin Leisure-Time Exercise Questionnaire, HCS = Health Contribution Score.

Similarly, patients were classified based on LVEF into normal, mildly reduced, and reduced LVEF as shown in Table 3. Accordingly, GLTEQ scores were 19.4 ± 14.4 for normal LVEF group, 27.7 ± 25.1 for mildly reduced group, and 16.8 ± 13.7 for the reduced LVEF group. Similarly, GLTEQ-HCS scores were 8.6 ± 14.5 units, 14.8 ± 23.4 units, and 7.2 ± 11.8 units for the normal, mildly reduced, and reduced LVEF groups, respectively. Relative grip strength was 1.1 ± 0.3 kg/BMI for both the normal and reduced LVEF groups and 1.2 ± 0.4 kg/BMI for the mildly reduced group as shown in Table 3.

**Table 3:** Participant differences in physical activity score, absolute grip strength, and relative grip strength as classified by cardiac performance based on left ventricle ejection fraction groups.

Outcome measure	LVEF category						*F* (2,93)	*η^2^*
	Normal		Mildly reduced		Reduced			
	M	SD	M	SD	M	SD		
Physical activity								
GLTEQ (units)	19.4	14.4	27.7	25.1	16.8	13.7	1.24	0.05
GLTEQ-HCS (units)	8.6	14.5	14.8	23.4	7.2	11.8	0.67	0.03
Absolute hand grip strength (kg)	31.5	9.1	33.2	7.9	29.4	8.3	2.59	0.03
Relative grip strength (kg/BMI)	1.1	0.3	1.2	0.4	1.1	0.3	1.47	0.01

GLTEQ = Godin Leisure-Time Exercise Questionnaire, HCS = Health Contribution Score, LVEF = Left Ventricle Ejection Fraction.

** *P* < .01.

## 4. Discussion

This study was aiming to describe the patterns and risk factors of CVD for patients admitted to PAMCC in Arar city, Saudi Arabia. Our findings showed that none of the tested variables (GLTEQ, GLTEQ-HCS, absolute hand grip strength, and relative hand grip strength) were significantly different when classified using the HBA1c diabetes status nor LVEF groups. Nonetheless, healthy participants (without DM) had higher GLTEQ-HCS mean score compared to the other groups. This observation supports the fact that participants with diabetes tend to avoid participating in physical activity.^[19,20]^ Similarly, although not significantly different, GLTEQ score by LVEF categories was approaching statistical significance (*P* = .080). This could also hint toward low physical activity levels being correlated with increased cardiovascular risk.^[21]^ This finding was evident by the majority of the participants scoring insufficiently active in GLTEQ (73.7%) compared to only 19.2% reporting being active according to GLTEQ classification.^[10]^ Furthermore, this may accord with a previous study where the reported norm for hand grip strength (absolute) in developing countries was 43.4 kg, which our sample scored lower than the reported norm.

The demographic data in the current study revealed that the majority of the participants had high school diplomas (83.0%), which may indicate that lack of access to higher education is linked to higher risk of CVD. One potential reason is that higher education is linked to lower consumption of sugar,^[22]^ knowing that increased sugar consumption is associated with increased risk of CVD.^[23]^ In addition, more than the majority of the participants (approximately 90%) reported living in a city with city life bringing more life stressors, which could raise the risk of CVD.^[24]^ Furthermore, smoking status revealed that more than two-thirds of the recruited sample were either current smokers or ex-smokers, confirming that smoking is a risk factor for CVD.^[25]^ Further, a previous study that examined the relationship between smoking and CVD in adults with CVD in Arar city in 2017 reported that out of 181 participants, 31.5% reported being nonsmokers, and only 22.1% reported being ex-smokers.^[26]^ This finding is consistent with a previous national study conducted in Saudi Arabia, which reported an increase in the percentage of adults using various tobacco products.^[27]^ This discrepancy indicates an increase in the proportion of current smokers and ex-smokers who had CVD in this region of Saudi Arabia in 2017. One could postulate that the concurrent smoking of other smoking options (e.g., electronic cigarettes and hookah smoking) may increase the risk of CVD among current smokers. To these ends, current policies for reducing and preventing smoking should be maintained. Future research should focus on smoking prevention strategies in Saudi Arabia.

Overall, more than a third (36.1%) of included participants had single vessel disease. Specifically, men had more severe CVD than women reflected by the higher proportion of triple vessel disease than women, whereas women had predominantly single vessel disease. This distribution is consistent with previous evidence suggests that men are more likely to have higher CVD vessel score, a score that reflects the number of blocked vessels, than women.^[28]^ In general, women have smaller coronary artery diameters, thereby, contributing the difference in affected vessels distribution.^[29]^ This was also consistent with an observed high total cholesterol levels and high triglycerides levels, a common predictor of CVD, in men of the studied sample. In the contrary, Altaleb and colleagues^[26]^ reported higher prevalence of CVD among women; notably, their study had 55.2% of their sample studied were females. This is the opposite of the current study.

Approximately two-thirds of our included sample had high income (>10,000 Saudi Riyal [SAR]/month). This observation is intriguing because having a high income may be associated with dietary patterns that include meat, dairy products, and refined starches. This shift has been shown to be related to increased cholesterol and triglyceride levels, which may increase the risk of CVD.^[30]^ We found no evidence that examined if individual income could be a risk factor of CVD in Saudi Arabia. This is important because higher individual income provides a variety of options for better health outcomes, such as access to safe environment to practice physical activity, lower financial-related stress, and higher education. However, the complexity of human behavior, increased demand on healthcare, and lack of time may play a role in increasing the risk of CVD, even among those of high income. Thus, further research is warranted to explore the association between individuals’ monthly income with CVD risk.

Our findings confirmed that increased levels of total cholesterol, LDL, triglycerides, and HbA1c are risk factors for CVD. This is in line with previous literature that reported that increase of these outcomes is considered risk factors for CVD development.^[31]^ However, there is limited evidence that validated these cardiovascular blood markers as risk factors for CVD in Saudi Arabia, leaving unexplored area of understanding CVD prevalence in this country. More efforts should be devoted towards identifying risk factors for CVD to achieve the national priority of reducing the rates of CVD. Registries and longitudinal study designs may help in creating a feeding source of information to understand the risk of CVD and eventually contribute to reducing its prevalence and risk.

Lower participation in moderate-to-vigorous physical activity has been noted across the sample. This was evident by the fact that approximately two-thirds of our included sample were classified as insufficiently active by GLTEQ-HCS scores. Evidence suggests that low physical activity levels is a modifiable risk factor for CVD.^[32]^ This is alarming due to the lack of proportion meeting the recommended volumes of moderate-to-vigorous physical activity and increased sedentary behavior and time spent on screen among people in Saudi Arabia.^[33]^ Furthermore, hand grip strength average scores, an important indicator of health outcomes, were lower than reported norm for developing countries (43.4 kg).^[34]^ These findings highlight the need for physical activity promotion and the importance of resistance exercise programs. Understanding the current knowledge and perception of the role of physical activity in physical health could enhance participation in physical activity and therefore reducing the risk of CVD in Saudi Arabia.

This study contributes to the existing literature by providing region-specific data on the cardiac profiles and risk factors of CVD patients in Arar city, Saudi Arabia. Specifically, our findings highlight consistently low physical activity levels and reduced handgrip strength among patients with CVD in Arar city. These observations emphasize the need for integrating structured physical activity counseling and resistance training into cardiac rehabilitation and secondary prevention programs in Saudi Arabia. Clinically, routine assessment of handgrip strength and leisure-time physical activity could be implemented as simple, low-cost indicators of functional capacity and cardiovascular risk in outpatient and inpatient settings. From a public health perspective, future research should focus on longitudinal studies with larger, multi-center cohorts to confirm these findings and explore causal pathways. Tailored interventions promoting physical activity and muscle-strengthening exercise should be evaluated for their effectiveness in improving cardiac outcomes. Furthermore, region-specific risk factor registries and patient-centered digital health tools may facilitate personalized CVD management and support the national Vision 2030 goal of extending life expectancy through preventive health strategies.

## 5. Study Limitations

This study has several limitations that should be acknowledged when interpreting its findings. Although multivariate analysis was considered, assumption testing indicated no significant associations between physical activity and the proposed predictors (age, BMI, HbA1c, LVEF, smoking status, and grip strength). Therefore, multivariate modeling was not pursued, as it would not have provided additional explanatory value in this dataset. In addition, the use of cross-sectional design limits the ability to establish causal relationships between the observed risk factors and cardiac outcomes. Furthermore, the study was conducted at a single cardiac center in Arar city, which may also limit the generalizability of the findings to other regions of Saudi Arabia. Additionally, the reliance on self-reported data for some variables, such as physical activity levels, introduces the possibility of recall bias. The lack of the inclusion of a control group is another limitation of this study. Finally, while we included a comprehensive set of variables in our analysis, there may be other unmeasured factors that could influence the cardiac profiles of CVD patients.

## 6. Conclusions

This study provides valuable insights into the cardiac profiles and associated risk factors of patients with CVD in Arar city, Saudi Arabia. The findings highlight the critical role of physical activity and handgrip strength in maintaining cardiac health, as well as the need for improved management of traditional cardiovascular risk factors such as hypertension, dyslipidemia, and DM. Public health strategies that promote physical activity and early detection of atherosclerotic cardiovascular disease through routine clinical assessments could be instrumental in reducing the burden of cardiovascular disease in this region.

## Acknowledgments

The authors extend their appreciation to the Deanship of Scientific Research at Northern Border University, Arar, KSA for funding this research work through the project number “NBU-FFR-2024-2508-06.” The author extends the appreciation to the Deanship of Postgraduate Studies and Scientific Research at Majmaah University for funding this research work through the project number (2084).

## Author contributions

**Conceptualization:** Mansour M. Alotaibi, Manal Alhowaish, Mohammed M. Alqahtani, Marzouq K. Almutairi, Abubakr S. Siddig, Amina A. Alenazi, Ansar S. Albanagi, Aisha S. Alenazi, Naif Z. Alrashdi.

**Data curation:** Mansour M. Alotaibi, Manal Alhowaish, Abubakr S. Siddig, Wajdan O. Alenazi, Amina A. Alenazi, Ansar S. Albanagi, Aisha S. Alenazi.

**Formal analysis:** Mansour M. Alotaibi, Mohammed M. Alqahtani, Marzouq K. Almutairi, Naif Z. Alrashdi.

**Funding acquisition:** Mansour M. Alotaibi, Naif Z. Alrashdi.

**Investigation:** Mansour M. Alotaibi, Anwar B. Almutairi, Manal Alhowaish, Mohammed M. Alqahtani, Abubakr S. Siddig, Wajdan O. Alenazi, Amina A. Alenazi, Ansar S. Albanagi, Aisha S. Alenazi.

**Methodology:** Mansour M. Alotaibi, Anwar B. Almutairi, Manal Alhowaish, Mohammed M. Alqahtani, Marzouq K. Almutairi, Abubakr S. Siddig, Wajdan O. Alenazi, Aisha S. Alenazi, Naif Z. Alrashdi.

**Project administration:** Mansour M. Alotaibi, Manal Alhowaish, Abubakr S. Siddig, Naif Z. Alrashdi.

**Resources:** Mansour M. Alotaibi, Manal Alhowaish, Abubakr S. Siddig, Naif Z. Alrashdi.

**Software:** Mansour M. Alotaibi, Mohammed M. Alqahtani, Naif Z. Alrashdi.

**Supervision:** Mansour M. Alotaibi, Anwar B. Almutairi, Marzouq K. Almutairi, Abubakr S. Siddig, Naif Z. Alrashdi.

**Validation:** Mansour M. Alotaibi, Naif Z. Alrashdi.

**Visualization:** Mansour M. Alotaibi.

**Writing – original draft:** Mansour M. Alotaibi, Anwar B. Almutairi, Manal Alhowaish, Mohammed M. Alqahtani, Marzouq K. Almutairi, Abubakr S. Siddig, Wajdan O. Alenazi, Amina A. Alenazi, Ansar S. Albanagi, Aisha S. Alenazi, Naif Z. Alrashdi.

**Writing – review & editing:** Mansour M. Alotaibi, Anwar B. Almutairi, Manal Alhowaish, Mohammed M. Alqahtani, Marzouq K. Almutairi, Abubakr S. Siddig, Wajdan O. Alenazi, Amina A. Alenazi, Ansar S. Albanagi, Aisha S. Alenazi, Naif Z. Alrashdi.

## Corrections

This article was originally published with an incorrect affiliation for Marzouq K. Almutairi. “Department of Physical Therapy, College of Medical Rehabilitation, Qassim University, Buraidah, Qassim Province, Saudi Arabia” has now been corrected to “Department of Physical Therapy, College of Applied Medical Sciences, Qassim University, Buraidah, Qassim Province, Saudi Arabia” in the online version.

This article was originally published with incorrect funding information. The funding number “NBU-FFR-2024-2508-XX” has now been corrected to “NBU-FFR-2024-2508-06.”
